# Where we stand on doxyPEP depends on where we sit: a viewpoint

**DOI:** 10.3389/fmicb.2025.1616111

**Published:** 2025-06-20

**Authors:** Thibaut Vanbaelen, Fabian Kong, Izumo Kanesaka, Sheeba Santhini Manoharan-Basil, Chris Kenyon

**Affiliations:** ^1^STI Unit, Department of Clinical Sciences, Institute of Tropical Medicine, Antwerp, Belgium; ^2^Centre for Epidemiology and Biostatistics, Melbourne School of Population and Global Health, University of Melbourne, Parkville, VIC, Australia; ^3^Department of Infection Control and Prevention, Faculty of Nursing, Toho University, Tokyo, Japan; ^4^Division of Infectious Diseases and HIV Medicine, University of Cape Town, Cape Town, South Africa

**Keywords:** doxyPEP, doxycycline, STI - science, chlamydia, gonorrhea

## Abstract

There is a striking variation in national doxycycline post exposure prophylaxis (doxyPEP) guidelines for sexually transmitted infections (STIs). Whilst some countries advocate doxyPEP for all men who have sex with men (MSM) and transgender women (TGW) with certain risks, others restrict the use to research settings. In this viewpoint, we argue that part of the explanation for this divergence can be attributed to different underlying conceptual frameworks. For individuals and organizations dominated by biomedical individualist frameworks, the primary goal of STI services is reducing the incidence of STIs. We have good evidence that doxyPEP does this and therefore, particularly in the setting of increasing STI incidence, this framework regards it as logical to roll out doxyPEP as fast as possible. By way of contrast, if organizations and their members operate within an ecosocial framework then their primary goal is the optimization of the sexual and overall health of individuals and populations and not just reducing STI rates. This framework sees the prevalence of STIs as being driven by the connectivity of local sexual networks. Recent increases in STI prevalence are seen as being due to increased network connectivity. The intensive use of antimicrobials such as doxycycline to reduce this prevalence is seen as introducing a selection pressure for the emergence of resistance to tetracyclines and other antimicrobials in *N. gonorrhoeae* and other species. This plus the other risks of doxyPEP, leads those animated by this framework to tend toward the precautionary principle and restrict the use of doxyPEP to research settings. The differences in these two frameworks thus leads different individuals and organizations with access to the same evidence-base to very different conclusions as to the net risk-benefit of doxyPEP.

## Background

Harrison et al. (1979), published the results of what remains the largest ever randomized controlled trial (RCT) to assess if tetracyclines taken post sex could reduce the incidence of gonorrhea. They found that minocycline post exposure prophylaxis (PEP) reduced the incidence of gonorrhea by 54% but advised against the use of minocycline PEP, due to the fact that it would select for tetracycline resistance. More specifically, they found that minocycline was 100% effective at preventing gonococcal infections with low tetracycline MICs but 0% effective against high MIC isolates. They concluded that “minocycline prophylaxis would probably have limited effectiveness as a public-health measure because of the tendency to select resistant gonococci (Harrison et al., 1979).”

More recent RCTs using doxycycline PEP (doxyPEP) to reduce the incidence of sexually transmitted infections (STIs) have had similar findings but reached the opposite conclusion. Three large RCTs in men who have sex with men (MSM) and transgender women (TGW) have found that doxyPEP reduces the incidence of chlamydia and syphilis by approximately 80% and the incidence of gonorrhea by up to 50% (Molina et al., 2018, 2024; Luetkemeyer et al., 2023, 2025; Szondy et al., 2024). These studies found that doxyPEP was associated with the emergence of tetracycline resistance in *N. gonorrhoeae*, commensal *Neisseria* species, *Staphylococcus aureus* and Group A *Streptococcus* (Luetkemeyer et al., 2023; Molina et al., 2024; Soge et al., 2025). There was also evidence compatible with the selection of methicillin resistant *S. aure*us) at an ecological level (Vanbaelen et al., 2024c). It should however, be noted that most concerningly was the evidence from the DOXYVAC study that the receipt of doxyPEP was associated with decreased susceptibility to cefixime in gonococci (Bercot et al., n.d.).

These findings would not have surprised the pioneers of antibiotic therapy such as Alexander Fleming who cautioned that the excessive use of antibiotics would select for antimicrobial resistance (AMR) (Rosenblatt-Farrell, 2009). In the subsequent century, a wealth of evidence has emerged to confirm this association and the utility of antimicrobial stewardship – reserving the use of antimicrobial therapy to instances where it is clearly necessary (Bell et al., 2014; Lawes et al., 2017; Spellberg, 2029). Systematic reviews of the short- and long-term use of tetracycline have clearly shown that, as with other classes of antimicrobials, tetracycline use selects for resistance to tetracyclines and other antimicrobials (Bell et al., 2014; Lawes et al., 2017; Truong et al., 2022; Vanbaelen et al., 2024a). That doxyPEP selects for AMR should thus come as no surprise. What is more surprising is that the authors of certain national doxyPEP guidelines have downplayed this concern. In Belgium, national guidance restricts the use of doxyPEP to research settings (De Scheerder et al., 2024). In contrast, the Centers for Disease Control and Prevention (CDC) guidelines in the United States advocate for the use of doxyPEP for all MSM and TGW who have had a bacterial STI in the past year as well to use a “shared decision-making approach” to discuss doxyPEP in other MSM and TGW (Bachmann et al., 2024). DoxyPEP guidelines in countries such as Australia, France, Germany, the Netherlands and the United Kingdom fall between these extremes (Saunders et al., 2021; Mårdh and Plachouras, 2023; Sherrard et al., 2024; Werner et al., 2024). The CDC guidelines note some of the associations between doxyPEP and AMR noted above, but entirely exclude others such as the data from Harrison et al. (1979) study. They conclude their guidance by noting that: “current data suggest overall benefit of the use of doxyPEP, but potential risks related to the development of resistance and changes in the microbiome will need to be monitored as these guidelines are implemented” (Bachmann et al., 2024).

How do we explain this divergence in interpretations of the risks and benefits of doxyPEP in national guidelines? In this perspective piece, we build on previous work to argue that this divergence of opinion about the net risk-benefit of doxyPEP stems in part from a difference in conceptual frameworks (Aral, 1999, 2001; Kenyon, 2020). More specifically it emerges from a difference in the conceptual framework we use to understand the determinants of STI spread and the relationship between antimicrobial consumption, STI prevalence, microbiomes, AMR and health. We also review the recent increases in STI incidence from a historical perspective to provide better context for this debate.

## The crucial need for explicit conceptual frameworks- where we stand depends on where we sit

Conceptual frameworks are crucial to structure our ideas and bring facts together in a way that they are able to form a coherent whole (Krieger, 1994; Susser and Susser, 1996; Kenyon et al., 2022). An optimal theory of the determinants of STI prevalence should thus provide an accurate portrayal of all the important determinants in a way that illustrates the interrelationships, the relative importance of the various determinants and facilitates proportionate and effective responses (Susser and Susser, 1996; Aral, 2001; Kenyon et al., 2022).

## Biomedical individualism and the argument to roll-out doxyPEP

**FIGURE 1:**
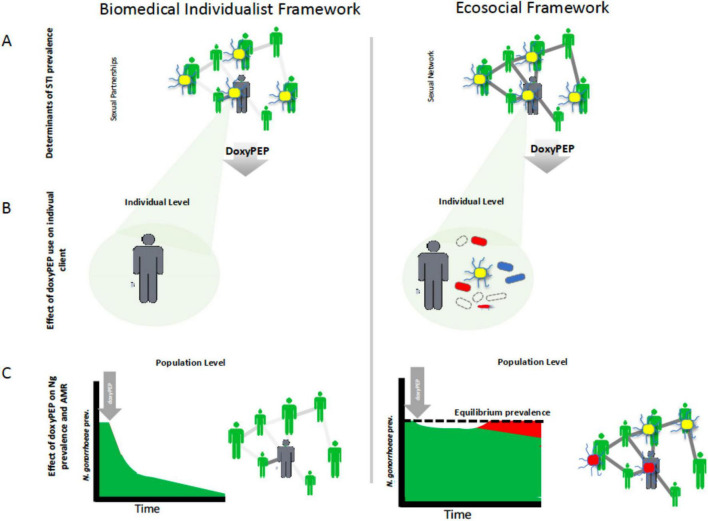
Schematic illustration of how different conceptual frameworks could lead researchers to divergent views on doxycycline postexposure prophylaxis (doxyPEP). The biomedical individualist framework views sexually transmitted infections (STIs) as obligate pathogens that can and should be eradicated by intensive seek-and-destroy activities **(A)**. It focuses on the individual and sees doxyPEP as an effective way to eliminate incident gonococcal infections (yellow diplococci) in individuals **(B)**. By rolling out doxyPEP to enough at-risk individuals it aims to eradicate *N. gonorrhoeae* and other STIs from a population **(C)**. By contrast, the ecosocial framework sees the high prevalence of STIs such as *N. gonorrhoeae* in certain populations as being a function of their denser sexual networks. Whilst doxyPEP may be reduce *N. gonorrhoeae* prevalence in the short term **(C)**, this lowers the prevalence of *N. gonorrhoeae* to below its equilibrium prevalence which in turn creates a selection pressure for the emergence of tetracycline resistance which will allow the gonococci to retain their equilibrium prevalence **(C)**. The doxyPEP will also select for tetracycline resistance in other bacteria [red oval bacteria in **(B)**] as well as resistance to other classes of antimicrobials [blue oval bacteria in **(B)**].

In previous work, we and others have discerned two dominant conceptual frameworks in the STI field (Aral, 2001; Kenyon, 2020; Kenyon et al., 2022). The biomedical individualistic conceptual framework has dominated the field for much of the past century (Hogben et al., 2020; Kenyon, 2020). This framework focuses on the individual patient and views STIs as obligate pathogens that can and should be eradicated by intensive seek-and-destroy activities (its primary goal) (Kenyon, 2020; Vanbaelen et al., 2025b). Elevated STI prevalences are seen as being primarily due to sexual behaviors and practices, as well as inadequate STI screening and treatment (Hogben et al., 2020; Kenyon, 2020; Kenyon et al., 2022). This framework assumes that STI prevalences can and should be brought to zero (Hogben et al., 2020; Kenyon et al., 2022). In settings of increasing STI prevalence doxyPEP is appealing to individuals operating within this framework, as doxyPEP has been proven to reduce the incidence of bacterial STIs (Bachmann et al., 2024; Figure 1).

The authors of the CDC doxyPEP guidelines argue that “increasing rates of bacterial STIs” and “the reported high efficacy” of doxyPEP were the main arguments for the decision to roll out doxyPEP (Bachmann et al., 2024). The authors note some concerns about AMR but conclude “systematic reviews of potential harms appear low in the short-term and unknown but potentially concerning in the long-term” (Bachmann et al., 2024). Despite the harms being “potentially concerning” in the long-term this does not stop the authors from actively promoting the roll-out of doxyPEP to a large population of MSM most at risk for gonococcal AMR (Bachmann et al., 2024). Individuals with the highest rates of partner turnover typically are at the highest risk for the emergence of AMR in *N. gonorrhoeae* and other STIs if heavily exposed to antimicrobials (Lewis, 2013; Kenyon and Schwartz, 2018). This has obvious risks for AMR. Certain proponents of doxyPEP have however, concluded that because doxyPEP would likely reduce exposure to ceftriaxone and azithromycin in this group, it would reduce selection pressure for AMR (Traeger et al., 2023).

## The ecosocial perspective and the argument to restrict doxyPEP to research settings

In contrast, the ecosocial framework is an explicitly multilevel framework that views monogamous and non-monogamous norms as equally ethical (Kenyon et al., 2022). STI prevalence is however, seen as largely a function of the connectivity of the local sexual network, and thus populations with high rates of partner turnover or concurrent partnering will have higher equilibrium prevalences of STIs (Kenyon and Delva, 2018; Kenyon et al., 2022). AMR in STIs is seen as typically emerging when populations with high STI prevalence are heavily exposed to antimicrobials – such as via intensive STI screening or doxyPEP (Lewis, 2013; Kenyon and Schwartz, 2018; Figure 1). The primary goal of this eco-social framework is optimizing the health of individuals and populations, which includes stewardship of their microbiomes and resistomes (Kenyon, 2020). A crucial aspect of this is using antibiotics in a way that optimizes cure rates while minimizing unnecessary/inappropriate use (stewardship) (Kenyon, 2020). One component of this approach is that “even in a patient with an obvious bacterial infection, one should only treat when therapy will alter the patient’s clinical course” and where the benefits clearly outweigh the harms (Spellberg, 2029). According to this approach *N. gonorrhoeae, C. trachomatis*, and *M. genitalium* infections in MSM that are usually asymptomatic and self-resolving are best classified as occasional pathogens that should be treated only when symptomatic (Kenyon et al., 2023; Vanbaelen et al., 2025b). As a result, physicians practicing within the ecosocial framework have questioned the utility of screening these three infections in MSM (Kenyon et al., 2023). This led them to first stop screening for *M. genitalium* in a PrEP cohort. This resulted in a 2- and 48-fold decline in macrolide and fluoroquinolone consumption, respectively (Kenyon et al., 2021, 2023). These encouraging results led them to then conduct an RCT to evaluate the effect of stopping screening for *N. gonorrhoeae/C. trachomatis* in MSM (Vanbaelen et al., 2024e). This RCT found that screening had little or no benefit but resulted in a large increase in antimicrobial consumption. These findings led to the cessation of screening in MSM on PrEP in Belgium (Van Praet et al., 2024). Because asymptomatic *N. gonorrhoeae/C. trachomatis* infections constitute the majority of the infections that doxyPEP prevents, and the ecosocial framework argues that these infections do not require therapy, the ecosocial views doxyPEP in a less favorable light that the biomedical individualistic framework (Vanbaelen et al., 2024f).

**FIGURE 2:**
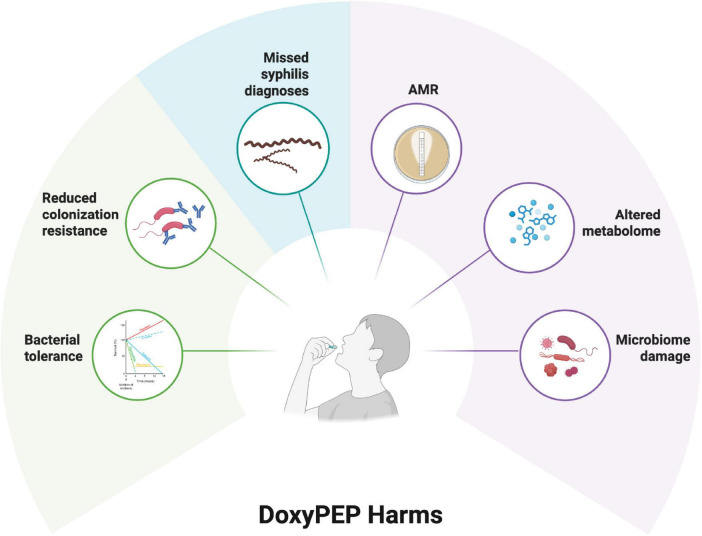
Putative harms of doxycycline postexposure prophylaxis (PEP) as summarized in six domains [see review by Kong et al. (2023)].

Whilst a range of arguments have been made to restrict doxyPEP to research settings (Figure 2), the most important argument is that doxyPEP will aggravate AMR (Kong et al., 2023). This could occur at both individual and population levels.

### Individual level

As noted above, the largest tetracycline PEP RCT thus far, found that minocycline PEP selected for tetracycline resistance in *N. gonorrhoeae* (Harrison et al., 1979). The more recent DOXYVAC RCT found that doxyPEP had a similar effect on gonococcal tetracycline resistance (Molina et al., 2024). The DoxyPEP RCT did not find this effect but the number of gonococcal isolates assessed was very small (Luetkemeyer et al., 2022, 2025). This study did, however, find that doxyPEP was associated with a higher prevalence of tetracycline resistance in commensal *Neisseria* spp. and *S. aureus* (Luetkemeyer et al., 2022, 2025; Vanbaelen et al., 2024a). This increase in *S. aureus* was however, not statistically significant (Vanbaelen et al., 2024a; Luetkemeyer et al., 2025). The DoxyPEP RCT was the only study to assess the impact of doxyPEP on the resistome using fecal metagenomic sequencing (Chu et al., 2024; Chu et al., 2025). This analysis revealed that doxyPEP had little or no effect on fecal microbiome but was associated with an increase in the abundance of genes conferring tetracyline resistance (Chu et al., 2024).

In a large number of species, including *N. gonorrhoeae*, tetracycline resistance is strongly associated with resistance to other antimicrobials (Gestels et al., 2023; Kenyon, 2024). By selecting for tetracycline resistance, doxyPEP could therefore inadvertently select for AMR to these other antimicrobial classes (Gestels et al., 2023). Tetracyclines have, for example, previously been shown to select for macrolide resistance in *Streptococcus pyogenes* (Nielsen et al., 2004). More recently, the DOXYVAC study found that doxyPEP was associated with an increase in gonococcal cefixime MICs (Bercot et al., n.d.; Vanbaelen et al., 2025a). This effect was mediated by selecting for strains with a mosaic *penA* allelle that has been linked to ceftriaxone resistance (Bercot et al., n.d.).

### Population level

The authors of the DOXYVAC study concluded that doxyPEP did not select for extended spectrum β-lactamse (ESBL) producing *Escherichia coli* or methicillin resistant *S. aureus* (MRSA) (Molina et al., 2024). They based this conclusion on the fact that there was no increase in the prevalence of these bacteria in the doxyPEP arm compared to the standard care arm (Molina et al., 2024). This analysis is however, limited by only considering individual level selection of AMR (biomedical individualist-based hypothesis testing). A number of analyses have established the importance of population-level selection of AMR (Lipsitch and Samore, 2002; Bell et al., 2014). As an example, differences in country level consumption of penicillin have been shown to explain approximately 80% of the variation in the prevalence of pneumococcal penicillin resistance in Europe (Goossens et al., 2005). Because the participants in both arms of the DOXYVAC study were interacting with one another, it is possible that doxyPEP in one arm could select for AMR in both arms (Vanbaelen et al., 2024c). A reanalysis of the DOXYVAC results using this ecological-hypothesis-testing, found that there was a significant increase in MRSA carriage in the doxyPEP arm (2%–12%) and a delayed and less pronounced increase in MRSA carriage in the standard care arm (2%–10%) (Vanbaelen et al., 2024c). These findings are compatible with the population-selection hypothesis (Vanbaelen et al., 2024c).

## The complicated interactions between network connectivity, STI prevalence, antimicrobial consumption and AMR

The evidence that AMR in STIs frequently emerges in sub-populations with dense sexual networks and excessive antimicrobial consumption, provides the rationale for the ecosocial framework to prioritize antimicrobial stewardship in these key populations (Lewis, 2013). It also necessitates an understanding of the links between network connectivity, STI prevalence, and AMR.

**FIGURE 3:**
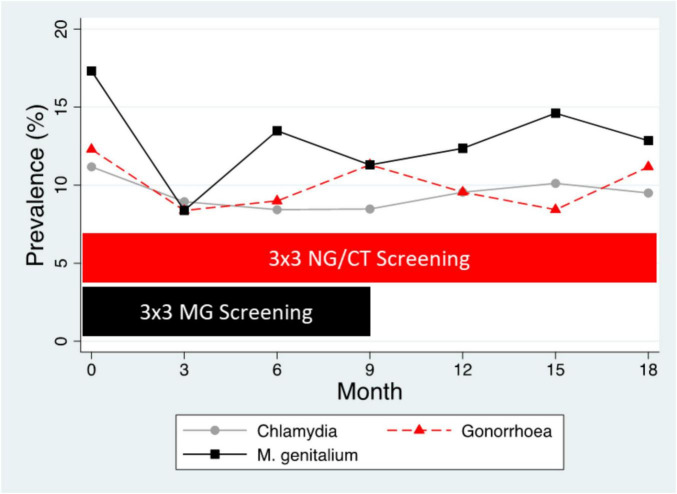
Prevalence of *N. gonorrhoeae* (NG), *C. trachomatis* (CT), and *M. genitalium* (MG) in the BePrEPared Study. This was an observational study of HIV PrEP in men who have sex with men who were tested for these three STIs at three-sites every 3 months. All infections were treated except those for *M. genitalium* after 9 months [3 × 3 screening– three site (anorectal, urethral, oropharynx), three monthly screening] (Reyniers et al., 2018).

Different types of evidence have established that sexual network connectivity is a fundamental determinant of the equilibrium prevalence of STIs and intense STI screening may have little effect on this prevalence (Kenyon and Delva, 2018; Kenyon, 2020). This is illustrated in Figure 3, which represents the *N. gonorrhoeae*, *C. trachomatis*, and *M. genitalium* prevalences of a typical PrEP cohort (Vuylsteke et al., 2019). The cohort report a mean of 5–10 partners per 3 months prevalence, which translates into a dense sexual network which in turn determines the high equilibrium prevalence of the three STIs of ∼ 10% (Vuylsteke et al., 2019). The introduction of three monthly screening for all three STIs at month 0 was perhaps associated with an initial reduction in STI prevalence but this effect was short lived (Vuylsteke et al., 2019). The cessation of *M. genitalium* screening at month 9 lead to a 50-fold decrease in fluoroquinolone consumption but not an increase in *M. genitalium* prevalence (Vuylsteke et al., 2019; Kenyon et al., 2021, 2023). A recent RCT has likewise concluded that three monthly screening of MSM taking PrEP had no effect on the incidence of *N. gonorrhoeae* (compared to non-screening) and a possible small effect on chlamydia incidence (Vanbaelen et al., 2024e). Both studies found that the vast majority of *N. gonorrhoeae, C. trachomatis*, and *M. genitalium* infections were asymptomatic and self-resolving (Vuylsteke et al., 2019; Vanbaelen et al., 2024e,2024d). Screening was associated with a large increase in antimicrobial consumption and various lines of evidence suggested that this was associated with the emergence of antimicrobial resistance (Kenyon, 2018; Kenyon et al., 2020; Van Dijck et al., 2020; Vanbaelen et al., 2024e).

**FIGURE 4:**
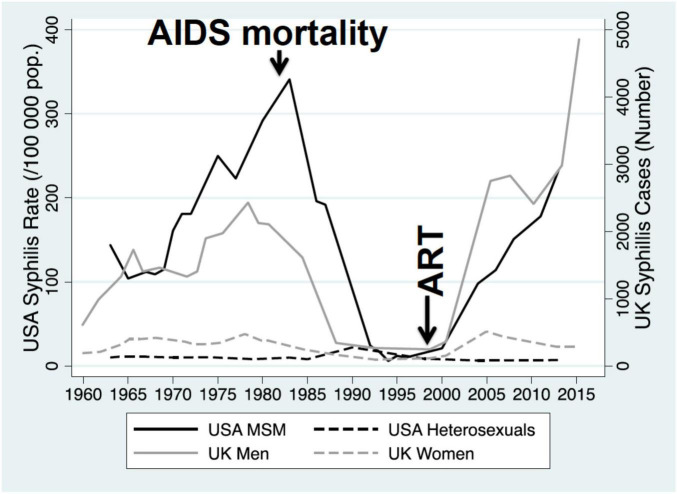
Incidence of primary and secondary syphilis among men who have sex with men (MSM) and heterosexuals in the United States, 1963–2013, and incidence of primary/secondary syphilis in men and women in the United Kingdom, 1960–2015. The AIDS epidemic and introduction of antiretroviral therapy (ART) are indicated with vertical arrows [modified from Kenyon (2020)].

**FIGURE 5:**
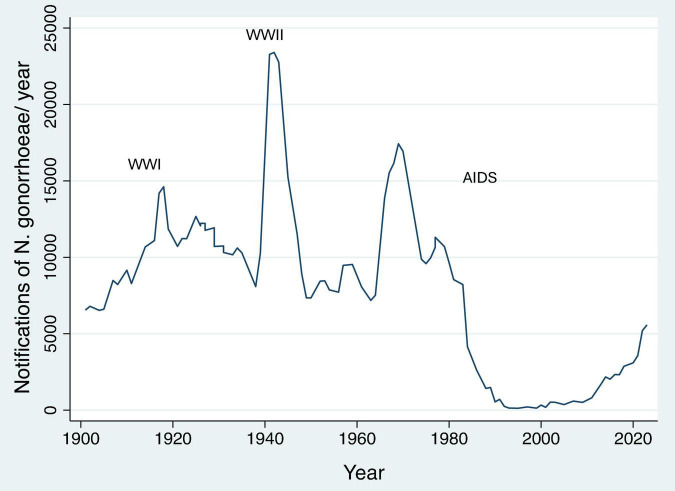
Number of *N. gonorrhoeae* notifications per year in Denmark between 1901 and 2023 [WWI/II – World War I/II; data from Statens Serum Institute (2023)].

Increases in network connectivity also offer a parsimonious explanation for the recent increases in multiple bacterial STIs noted in many countries (Kenyon and Delva, 2018; Kenyon et al., 2022). This is particularly evident if we consider data from the past 50 years. Using Denmark, the United States and the United Kingdom as examples, the incidence of bacterial STIs dropped precipitously in the 1980’s in response to the AIDS epidemic, which shattered sexual networks via behavior change and removing central nodes from the networks (Chesson et al., 2003; Figures 4, 5). The introduction of antiretroviral therapy for the treatment and prevention (PrEP) of HIV as well as other factors have been associated with an increase in sexual network connectivity (Chesson and Gift, 2008; Figures 4, 5). This has been followed by an increase in the incidence of syphilis, gonorrhea and other bacterial STIs (Mitjà et al., 2023). In the case of Denmark despite the dramatic increase in incidence in *N. gonorrhoeae* between 2020 and 2023 (46% in 2022 alone), the incidence remains below the pre-AIDS “equilibrium prevalence.” Seen from the ecosocial framework, this patterning would suggest the importance of not relying on the extremely low STI rates of the post-AIDS period (early 1990’s – Figure 4) as the baseline used for defining increases in STI incidence, which in turn justify doxyPEP rollout (Bachmann et al., 2024). This low STI incidence in the post-AIDS period (late 1980’s, early 1990’s) is depicted in Figure 4 where the incidence of syphilis in MSM in the United States fell to close to zero.

The ecosocial perspective highlights an important concern. Even if doxyPEP lowers the prevalence of *N. gonorrhoeae* and other bacterial pathogens in PrEP cohorts this is not necessarily a positive outcome, as it is from a biomedical individualist perspective. This is because if doxyPEP lowers the prevalence of *N. gonorrhoeae* below its equilibrium prevalence, then this will create a selection pressure for *N. gonorrhoeae* to acquire resistance conferring mutations that will enable to return to its equilibrium prevalence (Figure 1). The larger the decline in equilibrium prevalence the larger this risk will be. The same principles apply to other pathogens such as *S. aureus* whose prevalence has been reduced in certain doxyPEP RCTs (Luetkemeyer et al., 2023). We acknowledge that this concern is based on theoretical reasoning and the results of mathematical modeling and therefore requires empirical testing (Tsoumanis et al., 2023).

These considerations reveal a fundamental difference between the two perspectives. The biomedical individualist perspective aims for a maximum doxyPEP induced decline in STI prevalence and as such recommends targeting doxyPEP to those with the highest STI incidence such as MSM with the highest rates of partner turnover (Bachmann et al., 2024). The ecosocial perspective cautions that gonococcal AMR has typically emerged in key populations with high rates of partner turnover exposed to high antimicrobial consumption (Lewis, 2013). Targeting these same populations with intensive doxycycline, will once again place selection pressure for AMR emergence in these populations. This effect is congruent with that seen from other interventions involving the intensive use of antimicrobials through screening and mass treatment of STIs (Vanbaelen et al., 2024b). Particularly in MSM, these interventions result in at best a temporary decline in STI incidence at the expense of a long-term increase in AMR (Vanbaelen et al., 2024b). Similarly, previous attempts to use tetracyclines to prevent travelers’ diarrhea were halted by the emergence of AMR (Diptyanusa et al., 2018). A systematic review of the long-term use of tetracyclines for acne found a lack of high-quality evidence as to the risk of selection of AMR (Bhate et al., 2021). This review and other studies did however, find that long-term use of tetracyclines was associated with an increased risk of upper respiratory tract infections and pharyngitis. This effect could be explained by tetracycline-induced reduced colonization resistance (Kong et al., 2023). A more recent systematic review found that tetracycline use for various indications was associated with the selection of AMR (Truong et al., 2022).

The ecosocial perspective is therefore cautious about the use of antimicrobials to reduce STI prevalence in dense sexual networks. Rather it advocates for the use of vaccines, barrier contraception, condom use, and non-antibiotic compounds such as probiotics, chewing gums and bacteriophages as safer ways to reduce STI prevalence (Kenyon, 2020; Laumen et al., 2022; Adamczyk-Popławska et al., 2024). Noting the fact that most *N. gonorrhoeae* and *C. trachomatis* infections are asymptomatic and self-resolving in MSM, it is more inclined to a disease control approach that only tests and treats these STIs when they are symptomatic (van Bergen et al., 2021; Kenyon et al., 2023; Williams et al., 2023). In this sense it complies with the principles of optimal use of antimicrobial’s outlined by Spellberg (2029), such as limiting the use of antimicrobials to bacterial infections where treatment will alter the patient’s clinical course.

## Using doxyPEP to reduce syphilis incidence

Reducing the incidence of syphilis is the strongest argument for the use of doxyPEP for both perspectives. The Australian doxyPEP guidelines explicitly state that “doxy-PEP should be considered primarily for the prevention of syphilis in GBMSM” (Cornelisse et al., 2024). DoxyPEP is highly efficacious in this regard, involves little risk of inducing AMR in T. pallidum and the consequences of a missed syphilis infection can be severe (Kong et al., 2023; Cornelisse et al., 2024). The ecosocial perspective does however, note that doxyPEP used for syphilis prevention could still select for AMR in off target species. A further consideration is that doxyPEP may obscure the diagnosis of syphilis (Kong et al., 2023; Raccagni et al., 2024; Chircop et al., 2025). Treatment for syphilis with doxycycline requires 14–28 days therapy. Doxycycline taken intermittently may be sufficient to prevent the clinical signs of syphilis and prevent the normal serological response (Kong et al., 2023). This may result in missed or delayed diagnoses (Kong et al., 2023; Chircop et al., 2025).

## Community perspectives

For both perspectives it is crucial to take the opinions of the affected populations into account. A number of studies have confirmed that a high proportion of MSM are interested in using doxyPEP (Chow and Fairley, 2019; Evers et al., 2020; Hornuss et al., 2023). There is however, considerable concern about side effects (Evers et al., 2020). One study, for example, found that around 80% of the participants initially reported being willing to use doxyPEP, and 50% reported being concerned about side effects (Vanbaelen et al., 2025c). This study then provided participants with information about the risks of AMR. After receipt of this information, willingness to use doxyPEP decreased to 60% and concerns of side effects increased to 70%. These results suggest that the way individuals and communities view doxyPEP is influenced by how the intervention is framed and the net risk-benefit attributed to its use by experts.

## Conclusion

The available evidence shows that doxyPEP clearly reduces the incidence of bacterial STIs in MSM and TGW. Its impressive reduction in syphilis incidence could translate into large declines in the prevalence of symptomatic syphilis in not only this population but the general population as well (Szondy et al., 2024). It also clearly selects for AMR and some of the other deleterious outcomes listed in Figure 2. It is hard to combine these positive and negative outcomes into a single measure to evaluate the net cost-benefit of doxyPEP. One could try and calculate disability adjusted life years for these outcomes. This would however, to a great extent, involve assuming what these outcomes will be. This is because it is far from clear how quickly various STIs will rebound following rollout of doxyPEP and what the extent of doxyPEP-induced-adverse-effects such as AMR will be (Reichert and Grad, 2024). It has been the thesis of this manuscript that, in the absence of this information, individuals and organizations have fallen back onto their core ideologies. For those from a STI control (biomedical individualist) background where combating STIs is paramount, doxyPEP’s proven efficacy at reducing STIs makes it attractive for rapid roll out. For those from backgrounds such as infectious diseases and microbiology where ecological concepts and antimicrobial stewardship are foundational principles (Spellberg, 2029), the concerns of AMR have led to a more cautious approach. There are, of course, also a large number of other factors which could explain variations in attitudes to doxyPEP and therefore many exceptions to this claim are possible. It is also important to acknowledge that most individuals fall somewhere on the spectrum between the biomedical individualist and ecosocial approaches (Kenyon, 2020). Dividing individuals into more biomedical individualist and ecosocial frameworks does however, provide one way to help scientists understand how their colleagues may have very different opinions as to the rollout of doxyPEP. Ultimately, it is merely a hypothesis that requires empirical testing.

Whatever the reasons for the divergence in the rollout of doxyPEP, a benefit of this divergence is that it has set up a large natural experiment with some countries rolling out doxyPEP and others not. This should enable us to compare the individual- and population-level effects of doxyPEP in populations where it has and has not been rolled out. It would be useful to put more thought into collecting standardized samples and data from sentinel sites from these areas in a way that this comparison could be carried out in a meaningful way.

## Data Availability

The original contributions presented in this study are included in this article/supplementary material, further inquiries can be directed to the corresponding author.
